# Decreased Expression of Urinary Mammalian Target of Rapamycin mRNA Is Related to Chronic Renal Fibrosis in IgAN

**DOI:** 10.1155/2019/2424751

**Published:** 2019-08-14

**Authors:** Yuhan Cao, Yuwei Wang, Yinhua Liu, Xinjian Zhu, Guifa Zhang, Sufen Wang, Xiaomei Chen, Daoqin Liu, Cong Fu

**Affiliations:** ^1^Department of Nephrology, Yi Ji Shan Hospital Affiliated to Wannan Medical College, China; ^2^Key Laboratory of Non-Coding RNA Transformation Research of Anhui Higher Education Institution (Wannan Medical College), China; ^3^Department of Pathology, Yi Ji Shan Hospital Affiliated to Wannan Medical College, China; ^4^Department of Cardiology, Yi Ji Shan Hospital Affiliated to Wannan Medical College, China

## Abstract

**Background:**

Renal fibrosis is a common outcome of all pathological types of chronic kidney disease (CKD). However, the noninvasive detection of renal fibrosis remains a challenge.

**Methods:**

We collected urine samples from 154 biopsy-proven IgA nephropathy (IgAN) patients and 61 healthy controls. The expression of mTOR was measured and the correlation with renal function parameter and pathological indicators. The receiver operating characteristic (ROC) curve for the diagnosis of IgAN and renal fibrosis was calculated.

**Results:**

The urinary mammalian target of rapamycin (mTOR) expression was decreased in IgAN patients. The expression of mTOR was correlated with serum creatinine, blood urea nitrogen, estimated glomerular filtration rate, 24 h proteinuria, and cystatin C. Further, the urinary mTOR expression was significantly decreased in severe renal fibrosis patients compared with mild or moderate renal fibrosis patients. Urinary mTOR expression was correlated with score of tubulointerstitial fibrosis (TIF) and score of glomerular sclerosis. The ROC curve showed that mTOR can diagnose IgAN at a cut-off value of 0.930 with the sensitivity of 90.2% and specificity of 73.8% and renal fibrosis at a cut-off value of 0.301 with the sensitivity of 71.7% and specificity of 64.8%.

**Conclusion:**

Urinary mTOR mRNA expression was a potential biomarker for diagnosis of IgAN and renal fibrosis in IgAN patients.

## 1. Introduction

Chronic kidney disease (CKD) is a major public health problem worldwide and in China. The morbidity of CKD is 10.8% according to a cross-sectional survey [1]. IgA nephropathy (IgAN) is the major pathological type of CKD. The mechanism of IgAN is complicated that many factors such as abnormal IgA1 molecule [2], abnormal immune regulation [3, 4], podocyte injury [5], formation of fibrous scar in tubulointerstitium [6], hereditary factors [7], and environmental factors [8] participated in the progression of IgAN. Renal fibrosis, particularly tubulointerstitial fibrosis (TIF), is the common histological outcome of all types of CKD [9]. Accurate and early diagnosis of IgAN and renal fibrosis is important for treating and monitoring the progression of CKD.

The diagnosis of renal fibrosis, also the pathological progress, relies on renal puncture biopsy and pathological staining. Renal puncture is an invasive method which may result in bleeding and other serious complications [10]. Repeated renal biopsy is rare in the clinical practice that leads to difficult monitoring of disease progression. Classic biomarkers of CKD such as serum creatinine (Scr), blood urea nitrogen (BUN), and cystatin C (Cys-c) are incapable of accurately diagnosing renal fibrosis. Therefore, it is important to find a new noninvasive procedure and biomarkers which can diagnose renal fibrosis and pathological progression of CKD.

Recently, real-time quantitative polymerase chain reaction- (qPCR-) based urinary RNA detection has been developed for years as a novel strategy for the identification of renal kidney and CKD biomarkers [11–13]. Mammalian target of rapamycin (mTOR) is the key molecule that participated in cell proliferation, inflammation, and immunomodulation. Previous studies have indicated the relation between mTOR and CKD [14, 15]. However, if urinary mTOR RNA expression is related to renal fibrosis is unknown. Accordingly, this study was designed to determine the expression of urinary mTOR via qPCR to indicate renal fibrosis.

## 2. Methods

### 2.1. Study Design and Participants

A total of 154 biopsy-proven IgAN patients were selected from the Department of Nephrology, Yi Ji Shan Hospital, Wannan Medical College. Exclusion criteria are patients younger than 18 years old from 2017 to 2018; patients with chronic liver disease, urinary tract infection, cancer, or organ transplantation; signs or symptoms of severe complications, including cardiovascular disorder; or the use of steroids or immunosuppressive medications. Urine samples were collected 24 hours after a kidney biopsy, and clinical data were collected for all enrolled patients. Age- and gender-matched healthy volunteers (*n* = 61) from the Yi Ji Shan Hospital Health Care Center were also enrolled in the study as controls. This study was approved by the Ethical Committee of Yi Ji Shan Hospital, Wannan Medical College. Written informed consents were obtained from all subjects for the use of their urine and biopsy samples for research purposes.

### 2.2. Collection of Urine Samples and RNA Isolation

Whole stream early morning urine specimens were collected. Urine samples were centrifuged at 3,000 g for 30 min at 4°C. The remaining cell pellets were collected and then resuspended in 1.5 ml DEPC-treated PBS and centrifuged at 13,000 g for 5 min at 4°C. After being washed three times by diethyl pyrocarbonate- (DEPC-) treated phosphate buffer saline (PBS), the pellets were resuspended in 1.0 ml TRIzol Reagent (Ambion, Life Technologies) and stored at -80°C. Total RNA was extracted according to the manufacturer's protocol (Ambion, Life Technologies). Furthermore, the concentration and purity of RNA were assessed using the relative absorbance ratio at 260/280 in a NanoDrop 2000 (Thermo). 18S RNA was measured as the control.

### 2.3. Real-Time RT-qPCR

RT-PCR was performed using mTOR primers (sense: 5-TCCGAGAGATGAGTCAAGAGG-3; antisense: 5-CACCTTCCACTCCTATGAGGC-3) and 18S rRNA primers (sense: 5-CATGCTAACTAGTTACGCGACC-3; antisense: 5-GAGCAATAACAGGTCTGTGATG-3). After RT (50°C, 30 min), hot start (94°C, 15 min), and 40–42 cycles of PCR (94°C, 1 min; 52.5°C, 1 min; and 72°C, 1 min), mTOR mRNA expression was normalized to 18S rRNA and calculated as 2^−*ΔΔ*Ct^.

### 2.4. Assessment of Renal Fibrosis

Renal fibrosis was performed on paraffin-embedded sections stained with periodic acid-Schiff and Masson trichrome. Serial 3 m sections were acquired from each paraffin block. Two experienced pathologists who were blinded to the results of molecular studies subjectively scored the severity of renal fibrosis. Glomerulosclerosis was assessed in periodic acid-Schiff-stained sections using a semiquantitative scoring system according to the method of Schaier et al. [16]. Each glomerulus was graded from 0 to 4 according to sclerosis severity, and the average of all glomeruli in the entire tissue sample was calculated for analysis. The evaluation of the percentage of TIF was performed on Masson-stained sections and estimated the severity of TIF [17]. None was considered to be up to 5% of the renal interstitium, moderate between 26 and 50%, and severe > 50%. For analysis, biopsies with a TIF area < 25% were combined as none-to-mild fibrosis. Oxford histological classification was performed according to previous research [18, 19].

### 2.5. Statistical Analysis

SPSS 17.0 was used for data analysis. Relative changes in gene expression were calculated using the *ΔΔ*Ct (threshold cycle) method: ΔCt = Ct gene of interest‐Ct internal control, while ΔΔCt = (Ct gene of interest‐Ct internal control) sample‐(Ct gene of interest‐Ct internal control) control. Fold change = target gene expression level of sample/target gene expression level of control = 2^−ΔΔCt^. Normal distribution and equal variance data were compared using Student's *t*-test. A Mann–Whitney test was used for variance inequality or nonnormal distribution data. Spearman's rank-order correlation coefficient was used to assess associations between gene expression levels and clinical parameters. Stepwise multivariate logistic regression analysis including all univariate associates (*P* < 0.05) was used to assess the predictors for renal fibrosis. The diagnostic performance of biomarkers was evaluated using receiver operating characteristic (ROC) curves. The diagnostic threshold for maximum sensitivity and specificity was calculated. All *P* values were two-tailed, and *P* < 0.05 was considered statistically significant.

## 3. Results

### 3.1. Baseline Clinical and Pathological Characteristics

**Figure 1:**
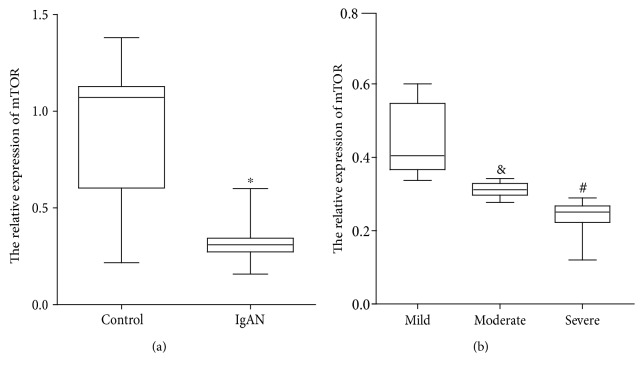
Urinary mTOR mRNA expression in IgAN patients and controls. (a) The relative expression of urinary mTOR in IgAN and healthy controls. (b) The relative expression of urinary mTOR in different degrees of renal fibrosis patients (^∗^*P* < 0.05 vs. control; ^#^*P* < 0.05 vs. mild and moderate; and ^&^*P* < 0.05 vs. severe).

**Figure 2:**
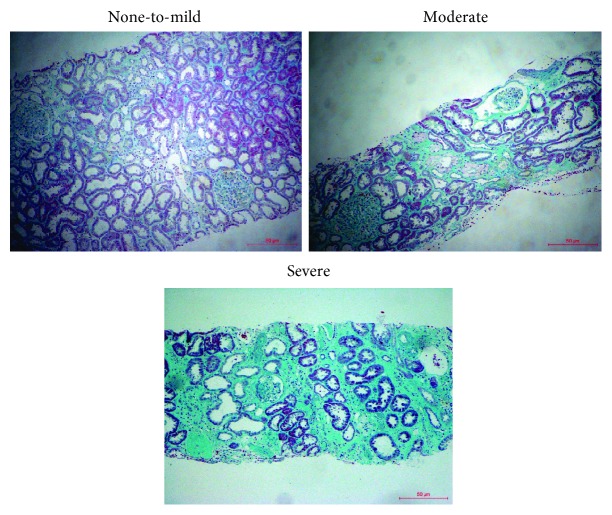
Representative histological findings of renal fibrosis stained by Masson's trichrome. None-to-mild fibrosis was considered as up to 25% of the tubulointerstitial fibrosis (TIF) area, moderate referred to an area 26%-50% of the TIF area, and severe referred to an area > 50% of the TIF area. Original magnification: ∗100.

Kidney biopsies were performed for the clinical diagnosis of IgAN. Primary clinical and pathological characteristics of the involved subjects are summarized in Table 1. There were no significant differences in age and gender between IgAN patients and controls. The IgAN group had a significant decrease in the estimated glomerular filtration rate (eGFR) compared with controls. eGFR was calculated using modified MDRD equations for Chinese patients [20]. Relative expression of mTOR was significantly decreased in the IgAN group (*P* < 0.05 vs. controls, Figure 1(a)). Oxford histological classification of IgAN patients was also shown in Table 1. Furthermore, the 154 IgAN patients were divided into 3 groups according to renal fibrosis degree. As shown in Table 2, there were no significant differences in age, gender, 24 h proteinuria, and BUN among the three groups. The eGFR in severe renal fibrosis was significantly lower compared with the other two groups. The relative expression of mTOR was significantly lower in moderate (*P* < 0.05 vs. none-mild) and severe fibrosis (*P* < 0.05 vs. moderate, none-mild) groups than none-mild fibrosis (Figure 1(b)).  Figure 2 showed the representative of different degrees of renal fibrosis confirmed by Masson trichrome.

### 3.2. Correlation between Urinary mTOR Expression, Clinical Parameters, and Renal Fibrosis

**Figure 3:**
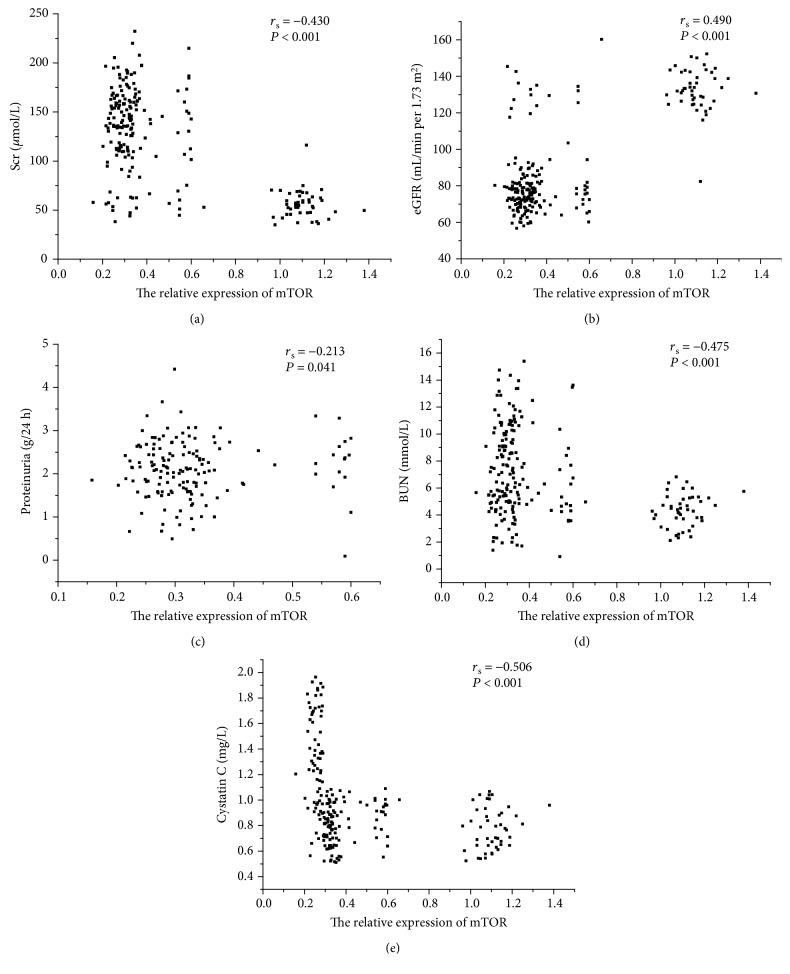
Correlation between urinary mTOR expression and clinical parameters. (a) Spearman correlation between mTOR expression and Scr (*r*_*s*_ = ‐0.430, *P* < 0.001). (b) Spearman correlation between mTOR expression and eGFR (*r*_*s*_ = 0.490, *P* < 0.001). (c) Spearman correlation between mTOR expression and 24 h proteinuria (*r*_*s*_ = ‐0.213, *P* = 0.041). (d) Spearman correlation between mTOR expression and BUN (*r*_*s*_ = ‐0.475, *P* < 0.001). (e) Spearman correlation between mTOR expression and cystatin C (*r*_*s*_ = ‐0.506, *P* < 0.001).

**Figure 4:**
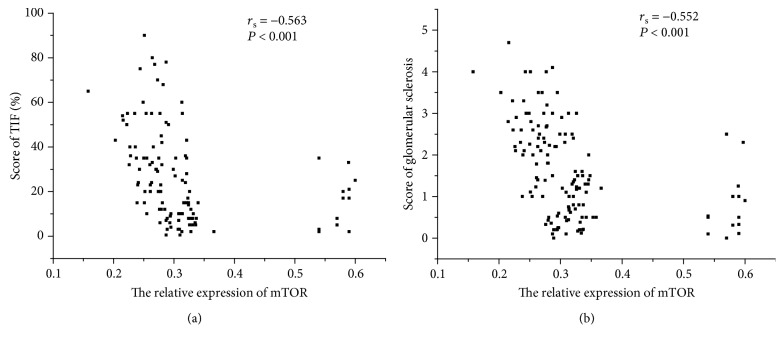
Correlation between urinary mTOR expression and renal fibrosis. (a) Spearman correlation between mTOR expression and score of TIF (*r*_*s*_ = ‐0.563, *P* < 0.001). (b) Spearman correlation between mTOR expression and score of glomerular sclerosis (*r*_*s*_ = ‐0.552, *P* < 0.001).

As shown in Figure 3, urinary mTOR mRNA levels correlated with Scr (*r*_*s*_ = ‐0.430, *P* < 0.001), BUN (*r*_*s*_ = ‐0.475, *P* < 0.001), eGFR (*r*_*s*_ = 0.490, *P* < 0.001), 24 h proteinuria (*r*_*s*_ = ‐0.213, *P* = 0.041), and Cys-c (*r*_*s*_ = ‐0.506, *P* < 0.001). Further, as shown in Figure 4, urinary mTOR mRNA levels correlated with score of TIF (*r*_*s*_ = ‐0.563, *P* < 0.001) and score of glomerular sclerosis (*r*_*s*_ = ‐0.552, *P* < 0.001).

Stepwise multivariate logistic regression analysis showed that the relative expression of urinary mTOR strongly correlated with the severity of renal fibrosis (Table 3, OR 10.325, 95% CI: 1.147-50.621, *P* < 0.001). The results indicated that the expression of urinary mTOR decreased every one unit; the risk for renal fibrosis elevated 10.358 times.

### 3.3. Diagnostic Value of Urinary mTOR mRNA Expression

**Figure 5:**
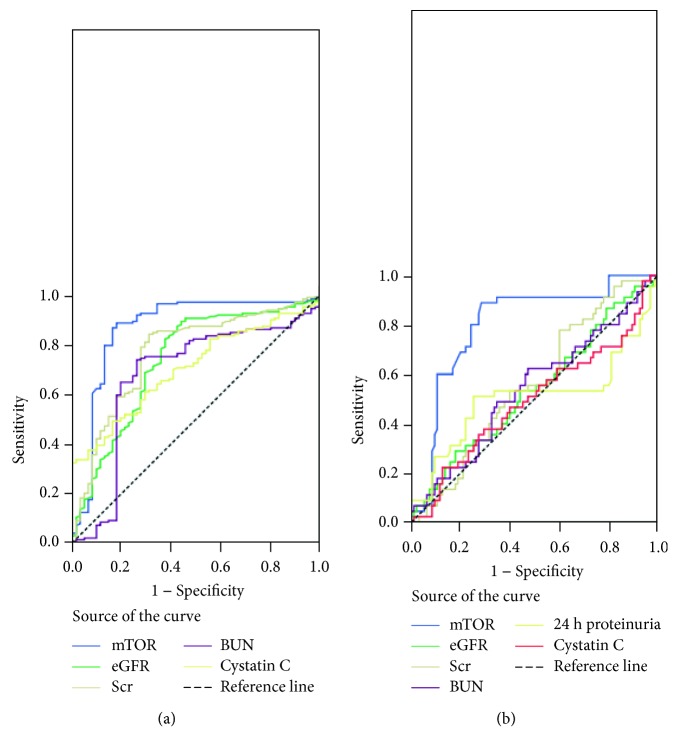
Receiver operating characteristic (ROC) curve showed the diagnosis value of urinary mTOR expression for IgAN and renal fibrosis. (a) ROC curve showed that the urinary mTOR mRNA level distinguished IgAN from controls (AUC = 0.868; 95% CI: 0.802-0.933; *P* < 0.001). (b) ROC curve showed that the urinary mTOR mRNA level distinguished moderate-to-severe fibrosis from none-mild fibrosis (AUC = 0.739; 95% CI: 0.654-0.824; *P* < 0.001).

The receiver operating characteristic (ROC) curve showed that the urinary mTOR mRNA level effectively distinguished IgAN from controls, with the largest AUC of 0.868 (95% CI: 0.802–0.933; *P* < 0.001), higher than that of eGFR (AUC of 0.738; 95% CI: 0.660–0.816; *P* < 0.001), Scr (AUC of 0.769; 95% CI: 0.697–0.84; *P* < 0.001), BUN (AUC of 0.618; 95% CI: 0.590–0.766; *P* < 0.001), and Cys-c (AUC of 0.704; 95% CI: 0.634-0.774; *P* < 0.001). mTOR displayed the sensitivity of 90.2% and specificity of 73.8% at the optimal cut-off value of 0.930 (relative gene expression level, Figure 5(a)).

Further, we evaluated the diagnostic value of urinary mTOR mRNA expression for renal fibrosis. The results showed that the urinary mTOR mRNA level effectively distinguished moderate-to-severe fibrosis from none-mild fibrosis, with the largest AUC of 0.739 (95% CI: 0.654-0.824; *P* < 0.001), higher than that of eGFR (AUC of 0.492; 95% CI: 0.395-0.588; *P* = 0.868), Scr (AUC of 0.513; 95% CI: 0.414-0.612; *P* = 0.794), BUN (AUC of 0.499; 95% CI: 0.400-0.598; *P* = 0.981), 24 h proteinuria (AUC of 0.510; 95% CI: 0.401-0.618; *P* = 0.850), and Cys-c (AUC of 0.495; 95% CI: 0.388-0.603; *P* = 0.055). mTOR displayed the sensitivity of 71.7% and specificity of 64.8% at the optimal cut-off value of 0.301 (relative gene expression level, Figure 5(b)).

**Figure 6:**
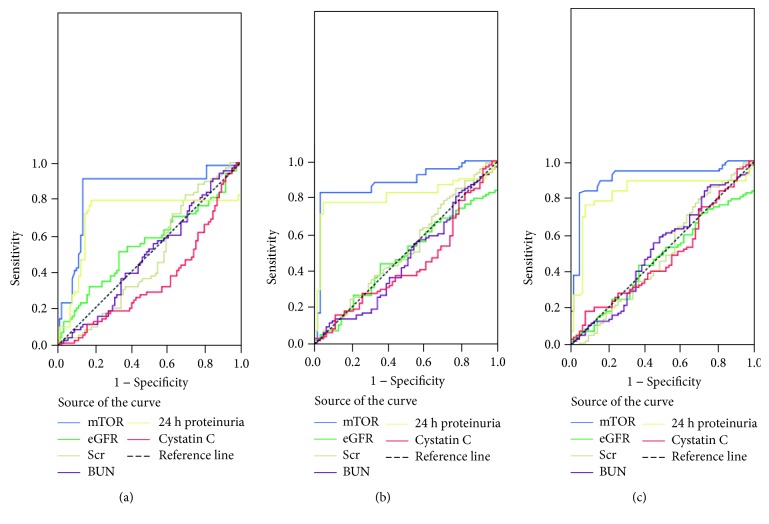
Receiver operating characteristic (ROC) curve showed the diagnosis value of urinary mTOR expression for E, S, and T scores. (a) ROC curve showed that the urinary mTOR mRNA level distinguished E1 from E0 (AUC = 0.841; 95% CI: 0.770-0.912; *P* < 0.001). (b) ROC curve showed that the urinary mTOR mRNA level distinguished S1 from S0 (AUC = 0.881; 95% CI: 0.823-0.940; *P* < 0.001). (c) ROC curve showed that the urinary mTOR mRNA level distinguished T2 from T0 and T1 (AUC = 0.909; 95% CI: 0.857-0.961; *P* < 0.001).

ROC curves for distinguishing E1 from E0, S1 from S0, and T2 from T0 and T1 were also performed. The results showed that the urinary mTOR mRNA level effectively distinguished E1 from E0 (Figure 6(a)), with the largest AUC of 0.841 (95% CI: 0.770-0.912; *P* < 0.001), higher than that of eGFR (AUC of 0.551; 95% CI: 0.457-0.644; *P* = 0.281), Scr (AUC of 0.476; 95% CI: 0.385-0.568; *P* = 0.617), BUN (AUC of 0.488; 95% CI: 0.396-0.580; *P* = 0.799), 24 h proteinuria (AUC of 0.707; 95% CI: 0.610-0.804; *P* = 0.051), and Cys-c (AUC of 0.362; 95% CI: 0.273-0.450; *P* = 0.063). mTOR displayed the sensitivity of 91.2% and specificity of 86.0% at the optimal cut-off value of 0.308. For S score (Figure 6(b)), the results showed that the urinary mTOR mRNA level effectively distinguished S1 from S0, with the largest AUC of 0.881 (95% CI: 0.823-0.940; *P* < 0.001), higher than that of eGFR (AUC of 0.476; 95% CI: 0.384-0.568; *P* = 0.614), Scr (AUC of 0.520; 95% CI: 0.425-0.614; *P* = 0.682), BUN (AUC of 0.462; 95% CI: 0.367-0.556; *P* = 0.424), 24 h proteinuria (AUC of 0.772; 95% CI: 0.689-0.884; *P* = 0.070), and Cys-c (AUC of 0.437; 95% CI: 0.343-0.530; *P* = 0.184). mTOR displayed the sensitivity of 82.8% and specificity of 90.7% at the optimal cut-off value of 0.312. For T score (Figure 6(c)), the urinary mTOR mRNA level effectively distinguished T2 from T0 and T1, with the largest AUC of 0.909 (95% CI: 0.857-0.961; *P* < 0.001), higher than that of eGFR (AUC of 0.468; 95% CI: 0.376-0.560; *P* = 0.505), Scr (AUC of 0.497; 95% CI: 0.401-0.594; *P* = 0.954), BUN (AUC of 0.516; 95% CI: 0.420-0.611; *P* = 0.740), 24 h proteinuria (AUC of 0.757; 95% CI: 0.655-0.860; *P* = 0.054), and Cys-c (AUC of 0.485; 95% CI: 0.391-0.578; *P* = 0.068). mTOR displayed the sensitivity of 82.6% and specificity of 90.2% at the optimal cut-off value of 0.313.

## 4. Discussion

Our study firstly indicated that urinary mTOR mRNA was a potential noninvasive biomarker of IgAN and especially chronic renal fibrosis. The discovery of CKD and renal fibrosis is a challenging topic attracting several of researchers' direction. The traditional biomarkers of CKD, for example, Scr, BUN, and eGFR, failed to reveal the pathological type of CKD. So far, there was a lack of reliable biomarkers of renal fibrosis, although many studies suggested some candidates [21, 22]. IgAN is a major pathological type of CKD. The previous study indicated that renal CD147 expression is a potential biomarker for IgAN [23]. Other researchers further screened tissue-specific microRNA expression in glomeruli and proximal tubules in IgAN patients [24]. However, renal biopsy was also the unique method of collecting kidney tissue which limited the application in the clinical practice. Repeated renal biopsy has a high risk level for patients. So far, there was no noninvasive method to identify the severity of renal fibrosis.

The urine contained an abundant biological message which can reflect the pathological changes. The changes of some renal fibrosis-associated molecule may reflect in urinary sediment cells, especially the podocyte and tubular epithelial cells. In recent years, qPCR was applicable in urinary mRNA biomarker detection. Li et al. [11] firstly established a noninvasive procedure to diagnose acute renal rejection of allografts by isolating and quantifying RNA of specific genes in urine cells. The obvious advance of qPCR such as high sensitivity and good repeatability makes qPCR a suitable noninvasive method to reflect kidney disease. Urinary sediment cell analysis was an appropriate procedure to screen novel mRNA biomarkers. Many previous studies indicated that urinary mRNA was a potential biomarker candidate of CKD [13, 25–27]. Zhou et al. further suggested that urinary mRNA expression showed favorable performance in diagnosing early renal fibrosis [28]. Our research revealed that urinary mRNA detection via qRT-PCR was a feasible method to identify the new biomarkers for IgAN and renal fibrosis. Urinary sediment was an appropriate source for IgAN and renal fibrosis diagnosis.

mTOR was a widely studied molecule which can regulate cell proliferation, inflammation reaction, and immune reaction [29, 30]. mTOR also participated in renal disease progression. Zhang et al. reported that mTOR signal pathways regulated immunosuppressive function in acute kidney disease [31]. In renal disease, mTOR has been identified as a potential therapy target [32]. Previous studies also indicated that mTOR played a key role in renal fibrosis [33, 34]. However, in different organs, microenvironments, and study objects, the role of mTOR showed an obvious heterogeneity. The key role of mTOR may act to adjust the balance of proinflammatory and anti-inflammatory responses [29]. In this study, we found that in IgAN patients' urinary sediment cells, mTOR mRNA expression was downregulated. Urinary sediment cells contain various types of cells such as podocyte, tubular epithelial cells, collecting duct cells, and even urethral epithelial cells. The downregulation of urinary mTOR mRNA may be a protective reaction in pathological status. The urinary mTOR expression level has diagnosis value for IgAN and renal fibrosis.

The previous studies showed that mTOR was considered as a biomarker of cancer, immune disease, degenerative diseases, and metabolic diseases [35, 36]. In IgAN, inhibition of mTOR ameliorates kidney injury [37]. It remained unknown if mTOR can act as a biomarker for IgAN, especially for renal fibrosis. Our study revealed that urinary mTOR mRNA expression was a potential biomarker for IgAN and renal fibrosis. Additionally, urinary mTOR mRNA expression also correlated with renal fibrosis in IgAN. Moreover, the ROC curve showed that urinary mTOR mRNA expression has potential to diagnose mesangial hypercellularity and endocapillary cellularity. A previous study indicated that the mTOR pathway has an important pathogenic role in diabetic nephropathy. However, if the mTOR signal pathway participated in the glomerular hypertrophy, then renal hyperplasia was uncertain [38]. Our research indicated that urinary mTOR mRNA can serve as a potential biomarker to diagnose renal fibrosis. It may be a potential noninvasive procedure that can identify IgAN and renal fibrosis.

In summary, our study demonstrated that detection of urinary mTOR mRNA could well predict renal fibrosis severity in IgAN, which suggested that this will serve as a novel independent noninvasive biomarker to monitor the progression of kidney fibrosis in IgAN.

Our study also has some limitations. Firstly, the current study is a discovery study focused on IgAN; if urinary mTOR expression can serve as a biomarker of renal fibrosis in other types of CKD needs to be further studied. Secondly, urethral epithelial cells in urinary sediment may influence the reliability of this method. Separation of different types of kidney cells can improve accuracy. Thirdly, to confirm the diagnostic value of urine mTOR mRNA in renal fibrosis and even the mesangial hypercellularity and endocapillary cellularity, a larger group of validation study and a long-term follow-up study are also necessary.

## 5. Conclusion

Urinary mTOR mRNA detection served as a noninvasive detection of IgAN and renal fibrosis. Urinary mTOR mRNA expression was a potential biomarker for diagnosis of IgAN and renal fibrosis in IgAN patients.

## Figures and Tables

**Table 1:** Clinical profile of patients with IgA nephropathy and healthy controls at the time of the kidney biopsy.

	IgAN (*n* = 154)	Control (*n* = 61)	*P* value
Age (years)	36.7 ± 1.6^a^	35.6 ± 2.1^a^	0.097^c^
Gender (male/female)	86/68	37/24	0.157^d^
Proteinuria (g/day)	2.1 ± 0.7^a^	n.d.	—
Scr (mmol/l)	146.8 ± 35.4^a^	54.9 ± 9.8^a^	<0.001^c^
BUN (mmol/l)	7.8 ± 3.6^a^	4.2 ± 1.1^a^	<0.001^c^
Cystatin C (mg/l)	1.27 ± 0.7^a^	0.68 ± 0.2^a^	<0.001^c^
eGFR (ml/min per 1.73 m^2^)	74.5 ± 8.3^a^	131.8 ± 10.2^a^	<0.001^c^
SBP (mmHg)	138.1 ± 6.4^a^	121.6 ± 7.9^a^	<0.001^c^
DBP (mmHg)	89.8 ± 7.6^a^	76.4 ± 5.6^a^	0.026^c^
Relative mTOR expression	0.412 (0.158-0.597)^b^	1.147 (0.217-1.380)^b^	<0.001^e^
Usage of ACEI/ARB	96	n.d.	—
Usage of diuretic	83	n.d.	—
Oxford histological classification (number)			
M score (0/1)	0/154	n.d.	—
E score (0/1)	80/74	n.d.	—
S score (0/1)	61/93	n.d.	—
T score (0/1/2)	62/65/27	n.d.	—
C score (0/1/2)	145/7/2	n.d.	—

^a^Data are presented as means (±SD). ^b^Data are presented as the median (min, max). ^c^Student's *t*-test. ^d^*χ*^2^ tests. ^e^Mann–Whitney test. Abbreviations: Scr: serum creatinine; BUN: blood urea nitrogen; eGFR: estimated glomerular filtration rate; SBP: systolic blood pressure; DBP: diastolic blood pressure; M: mesangial hypercellularity; E: endocapillary hypercellularity; S: segmental glomerulosclerosis; T: tubular atrophy/interstitial fibrosis; n.d.: not determined.

**Table 2:** Clinical and pathological parameters of patients with IgAN with different renal fibrosis degrees.

	None-mild fibrosis (*n* = 64)	Moderate fibrosis (*n* = 54)	Severe fibrosis (*n* = 36)	*P* value
Age (years)	34.25 ± 9.13^a^	39.39 ± 16.55^a^	40.54 ± 9.38^a^	0.254^c^
Gender (male/female)	30/34	33/21	19/17	0.526^d^
Proteinuria (g/day)	1.8 ± 0.6^a^	2.1 ± 1.9^a^	2.3 ± 1.8^a^	0.125^c^
Scr (*μ*mol/l)	60.26 ± 15.15^a^	179.28 ± 24.45^a^	187.74 ± 42.35^a^	0.021^c^
BUN (mmol/l)	8.9 ± 1.8^a^	9.2 ± 3.7^a^	10.8 ± 3.2^a^	0.074^c^
Cystatin C (mg/l)	0.75 ± 0.14^a^	1.21 ± 0.27^a^	1.53 ± 0.43^a^	0.042^c^
eGFR (ml/min per 1.73 m^2^)	96.32 ± 18.78^a^	64.36 ± 23.85^a^	50.24 ± 19.24^a^	<0.001^c^
SBP (mmHg)	121.56 ± 14.71^a^	142.83 ± 20.73^a^	157.15 ± 30.69^a^	<0.001^c^
DBP (mmHg)	80.56 ± 16.39^a^	82.41 ± 20.15^a^	93.21 ± 14.24^a^	0.018^c^
Usage of ACEI/ARB	51	32	13	0.067^d^
Usage of diuretic	38	35	10	0.053^d^
Relative mTOR mRNA expression	0.427 (0.382-0.597)^b^	0.312 (0.276-0.341)^b^	0.223 (0.158-0.289)^b^	0.024^e^
Score of glomerular sclerosis	0.5 (0-0.8)^b^	1.8 (0.5-3.0)^b^	2.5 (2.0-4.8)^b^	<0.001^e^
Score of TIF (%)	5 (0-4)^b^	30 (27-51)^b^	61 (52-90)^b^	<0.001^e^

^a^Data are presented as means (±SD). ^b^Data are presented as the median (min, max). ^c^Student's *t*-test. ^d^*χ*^2^ tests. ^e^Mann–Whitney test. Abbreviations: Scr: serum creatinine; eGFR: estimated glomerular filtration rate; SBP: systolic blood pressure; DBP: diastolic blood pressure; TIF: tubulointerstitial fibrosis.

**Table 3:** Multivariate logistic regression analysis of selected variables for TIF severity.

	OR	95% CI	*P* value
mTOR	10.358	1.147-50.621	<0.001
Scr	0.513	0.245-2.381	0.225
BUN	0.425	0.112-3.651	0.307
Cystatin C	0.624	0.159-4.215	0.521
eGFR	1.834	1.025-4.021	0.031
24 h proteinuria	1.657	0.567-3.441	0.104

Abbreviations: Scr: serum creatinine; eGFR: estimated glomerular filtration rate; OR: odds ratio.

## Data Availability

The data used to support the findings of this study are available from the corresponding authors upon request.
